# Fearful Foragers: Honey Bees Tune Colony and Individual Foraging to Multi-Predator Presence and Food Quality

**DOI:** 10.1371/journal.pone.0075841

**Published:** 2013-09-30

**Authors:** Ken Tan, Zongwen Hu, Weiwen Chen, Zhengwei Wang, Yuchong Wang, James C. Nieh

**Affiliations:** 1 Key Laboratory of Tropical Forest Ecology, Xishuangbanna Tropical Botanical Garden, Chinese Academy of Science, Kunming, China; 2 Eastern Bee Research Institute, Yunnan Agricultural University, Kunming, China; 3 Division of Biological Sciences, Section of Ecology, Behavior, and Evolution, University of California San Diego, La Jolla, California, United States of America; Arizona State University, United States of America

## Abstract

Fear can have strong ecosystem effects by giving predators a role disproportionate to their actual kill rates. In bees, fear is shown through foragers avoiding dangerous food sites, thereby reducing the fitness of pollinated plants. However, it remains unclear how fear affects pollinators in a complex natural scenario involving multiple predator species and different patch qualities. We studied hornets, *Vespa velutina* (smaller) and *V. tropica* (bigger) preying upon the Asian honey bee, *Apis cerana* in China. Hornets hunted bees on flowers and were attacked by bee colonies. Bees treated the bigger hornet species (which is 4 fold more massive) as more dangerous. It received 4.5 fold more attackers than the smaller hornet species. We tested bee responses to a three-feeder array with different hornet species and varying resource qualities. When all feeders offered 30% sucrose solution (w/w), colony foraging allocation, individual visits, and individual patch residence times were reduced according to the degree of danger. Predator presence reduced foraging visits by 55–79% and residence times by 17–33%. When feeders offered different reward levels (15%, 30%, or 45% sucrose), colony and individual foraging favored higher sugar concentrations. However, when balancing food quality against multiple threats (sweeter food corresponding to higher danger), colonies exhibited greater fear than individuals. Colonies decreased foraging at low and high danger patches. Individuals exhibited less fear and only decreased visits to the high danger patch. Contrasting individual with emergent colony-level effects of fear can thus illuminate how predators shape pollination by social bees.

## Introduction

The impacts of predation cascade through an ecosystem: predators can influence prey and thus affect primary producers [1]–[3]. Predator effects on pollinators are particularly important. Over 90% of flowering plant species in terrestrial ecosystems use animal pollinators to assist their reproduction [4], and 67% of flowering plants use insect pollinators [5]. Indirect top-down effects, mediated by predation of pollinators may therefore be common [6] and have strong ecosystem effects [3]. Predation can directly reduce pollinator numbers, but also exerts an important non-lethal effect, fear, which alters prey spatial distribution and foraging frequency [7]–[10]. Fear results from the anticipation or awareness of danger [11]. We use a functional definition of “fear” as prey exhibiting wariness and avoiding a predator [12].

The ecological consequences of fear can be as strong as actual predator consumption [13]. Fear is an effective pollinator deterrent, disrupting plant pollination and thereby affecting plant fitness. Crab spider presence, for example, resulted in fewer pollinator visits for shorter durations and decreased a measure of plant fitness, seed production in *Leucanthemum vulgare* [6]. Artificial crab spider models that could not kill insects similarly reduced insect pollinator visits to *Rubus rosifolius* flowers, resulting in 42% reduced seed set and 50% fruit mass decrease [14]. Fruit production of western monkshood, a bumble bee pollinated plant, significantly decreased at sites with high beewolf hornet activity [15]. In this case, 32% of attacks ended in successful predation, but, overall, studies demonstrate that predator effects are largely non-consumptive and result from fear of predators [16].

Studies that examine the effects of multiple predators upon prey behavior remain less common than single-predator studies. To understand how prey manage multi-level risk [17], we therefore need more data on how prey show vigilance [18] to multiple predators. Researchers studying pollinators have generally examined responses to a single predator species at a time [14], [19]–[24]. However, animals often face danger from multiple predator species that are differentially dangerous.

We focused on bees because they are important pollinators in a wide variety of ecosystems [25], influence plant fitness [6], [14], [15], are prey for multiple predators [15], [22], [26], and exhibit anti-predator avoidance. Bumble bees (*Bombus ternarius*) visited milkweed patches with spiders at a significantly lower rate compared to patches without spiders [20]. Honey bees (*Apis mellifera*) preferred safe over dangerous feeders (with a dead bee or a dead spider) and avoided revisiting sites where they experienced a predation attempt [21]. Honey bees also avoided flowers with crab spiders and flowers that had recently held spiders [22]. Dukas and Morse [19] compared pairs of milkweed patches and found that *A. mellifera* visited spider-infested patches less often, though not when natural spider densities were low [27]. However, it is not known how social bees will respond when presented with a naturally occurring situation of multiple predator species corresponding to different danger levels at food patches. Such information is important because understanding the size and strength of indirect effects in trophic cascades requires detailed knowledge of how prey respond to predators [28].

Bee predation studies have focused on sit-and-wait predators, like crab spiders, that wait on an inflorescence for pollinators. However, aerial predators may also be important and have a larger hunting area per predator. For example, Wilson and Holway [29] provided evidence that wasp (*Vespula pensylvanica*) presence elicited avoidance in *Hylaeus* bees. The “beewolf” wasp (*Philanthus* spp.) preys only on bees and its presence reduces bumble bee abundance and monkshood fruit set within a 50 km^2^ area around a hornet aggregation [15]. Hornets can also affect bee pollination by directly competing for nectar resources. Bumble bees avoid competing *Vespula* hornets on milkweed flowers [30]. *Vespula pensylvanica* presence reduces floral visitation by bees and decreases fruit set [31]. The effects of aerial predators like hornets upon bee foraging and pollination therefore deserves greater attention.

**Figure 1:**
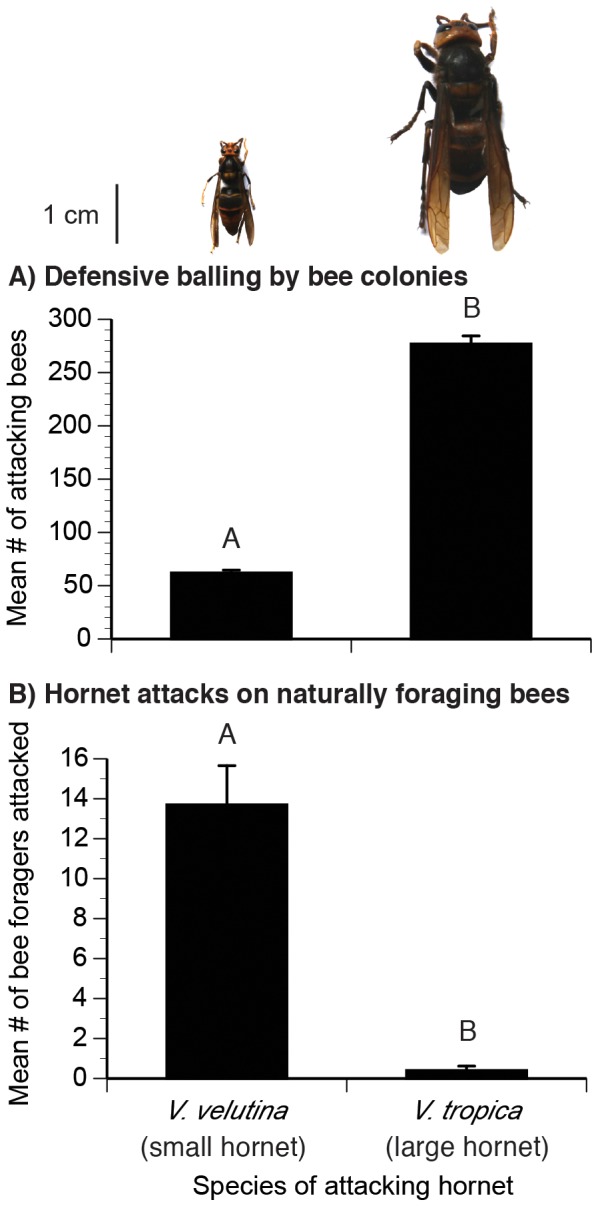
Hornets are a threat. Hornets (scaled photos shown) are (A) attacked by *A. cerana* colonies and (B) attack *A. cerana* foraging on natural flowers. Standard error bars are shown. We indicate significant differences with different letters.

Finally, fear-driven systems are traditionally described in terms of fierce vertebrate carnivores, in which fierceness is measured by prey responses [8]. The bee-hunting hornets are also fierce predators and are important because they prey upon key pollinators. In Asia, hornets within the genus *Vespa* are major honey bee predators and can lead to colony attrition and absconding [32], [33]. The big Asian hornet, *Vespa tropica*, will rob honey by first killing guard honey bees until the colony absconds [32]. Seeley et al. [34] reported that *V. tropica* could cause an *A. florea* colony of approximately 6000 bees to abscond after just three hours of fighting. The hornet, *V. velutina*, although smaller in body size than *V. tropica*, is similarly damaging [26], [35], [36]. Researchers have previously studied these hornets as honey bee nest predators, but we also observed them flying over flowers hunting for foraging *A. cerana*. Because *V. tropica* is four-fold more massive than *V. velutina* (Fig. 1), we predicted it would present a greater threat.

The Asian honey bee species, *A. cerana*, evolved with these *Vespa* predators and therefore has special defenses: a shimmering behavior that repels hornets [26], [37] and, as a final defense, surrounding the well-armored hornet with a ball of bees that kill with their intense body heat [38]. *Apis cerana* is also an important native pollinator [39], [40]. We therefore chose this species to test the hypothesis that a valuable pollinating insect would exhibit differential vigilance when presented with multiple food patches simultaneously containing different predator species. Bee predators can prefer patches containing high quality food that is more attractive to prey [41], [42]. In one experiment, we therefore matched food quality to danger, testing how bees respond when given choices among patches that were higher in quality (more concentrated nectar) and also higher in danger.

We measured both colony- and individual-level responses because honey bees are superorganisms and thus foraging decisions occur at individual and colony levels [43]. Like *A. mellifera*, *A. cerana* can recruit nestmates to floral resources [44], thus potentially amplifying the effects of vigilance at the colony level. For example, *A. mellifera* recruit less for a nectar resource that is perceived to be dangerous when a recently dead bee is placed on the resource [45].

## Materials and Methods

We conducted experiments from July–December 2012, corresponding to the period of peak hornet activity, at Yunnan Agricultural University, Kunming, China (22°42′ 30 N, 100°56′01 E, 1890 m altitude) when *V. velutina* and *V. tropica* were actively hunting honey bees. Both hornet species occur throughout Southeast Asia [36]. In Nepal, *V. velutina* and *V. tropica* attacks on *A. cerana* are similarly elevated from July through September [46]. Our field season also corresponded to a period of floral dearth, which facilitated feeder training of bees. We used three colonies of *A. cerana*, each with four frames of bees and brood.

### Heat-balling experiment

We first tested if *A. cerana* would treat *V. velutina* and *V. tropica* workers as a threat. Although *A. cerana* will attack and heat-ball *V. velutina* [26], [37], no published studies report on how it responds to *V. tropica*. We used insect nets to capture *V. velutina* and *V. tropica* workers in the apiary when they were hunting honey bees, anesthetised them with CO_2_, weighed each hornet, and then tethered each live hornet by wrapping the end of a wire (2.2 mm diameter and 50 cm long) around its waist (connection between thorax and abdomen) for presentation to bee colonies. We observed the response of naïve *A. cerana* workers to hornet presence (15 hornets of each species, five trials conducted on different days with each of three different *A. cerana* colonies, each bee tested only once). Each trial consisted of a living tethered hornet placed 10 cm away from the colony entrance. After 3 min, when the bees attacked, we placed the resulting bee ball into a sealed plastic bag, chilled it at 0°C for two minutes to kill the bees (*A. cerana* is a tropical species that is more sensitive to cold than *A. mellifera*), and then counted them.

### Hunting on flowers

To determine if hornets would naturally hunt bees on flowers, we observed a 1.5 m×2 m patch of *Cuphea balsarnona* flowers visited by *A. cerana* foragers. We counted the number of *V. velutina* and *V. tropica* hornets attempting to capture (diving down upon) *A. cerana* foragers in this patch for a 30 min trial (10 total trials, each on a different day). We also counted the number of successfully captured bees.

### Choice experiments

We conducted three different experiments defined by choices given to bees. We trained *A. cerana* foragers to an artificial feeder (70 mL vial with 18 holes of 3 mm diameter drilled around its lid) filled with unscented 30% sucrose solution, 10 m from the colony to be tested. We trained bees by capturing departing foragers at the hive entrance and releasing them slowly at the training feeder. Some landed, fed, and returned to their hive where they recruited nestmates.

At the start of each trial, we removed the training feeder and set out three feeders spaced 30 cm apart and equidistant from the focal colony. We again used unscented sucrose solutions for all experiments. Live insects (hornets or the butterfly, *Papilio xuthus*, a harmless control) were attached as described above, each to a wire and positioned 10 cm above their respective feeders. Each experiment consisted of two parts: (1) testing colony labor allocation among the feeders (group behavior) and (2) individual forager choices tested in the absence of other bees around the feeder array to ensure independent choices.

To measure the *colony labor allocation*, we set out the feeder array once 50 bees had been trained to the training feeder. Each feeder had a different color card (yellow, blue, or green) randomly assigned at the start of each trial and changed for the next trial. The location of each treatment within the array was also randomized between trials. At the array, we counted the number of bees on each feeder after 15 min, adequate time for colonies to recruit 2.7 fold more foragers, on average, than were trained. We predicted that colonies would allocate this labor according to the degree of risk and food quality. We also measured the feeding duration of 10 different randomly selected bees at each feeder successively from three colonies (30 bees per colony, three different feeders, total of 90 bees). We predicted that bees would spend less time feeding at risky feeders, according to the degree of risk and food quality.

To test *individual bee choices*, we trained a new set of bees to a set of three feeders also placed 10 m away from the subject nest, but in a different direction, and inside a caged arena (70×66×52 cm). This allowed us to control bees entering the arena and limit choices to one bee at a time. In the *learning phase*, each feeder was placed on a conspicuously colored card (yellow, blue or green) so the bee could associate the treatments at the different feeders with the card colors. This association was important because we subsequently monitored 10 successive bee choices and wanted the bees to easily detect which feeder corresponded to which treatment. Honey bees have excellent color vision and can associatively learn color [47]. To prevent potential color bias from influencing our results, we randomized color associations. Different bees were trained to associate a different series of colors with the three treatments. For example, the first bee was trained to associate green, blue, and yellow with treatments 1, 2, and 3 respectively. We then tested this bee with the same color associations. However, the second bee was trained to associate a different ordering of colors (blue, green, and yellow) with treatments 1, 2, and 3 respectively. We marked all trained foragers with individually numbered honey bee queen tags (Opalith-Zeichenplättchen) attached to their thoraces with resin glue.

Training was complete when the bee visited each feeder at least 10 times (approximately two training days per bee). We then added the treatment (blank control, live butterfly, or tethered live hornet) to each feeder (see below), and observed the trained bee for 10 visits to the array. A choice is a bee landing and feeding on a feeder. The bee was allowed to feed, leave the cage, and return for a total of 10 visits. After each visit, we randomly swapped feeder positions to avoid potential site bias. We trained 15 bees from each of three colonies, testing a total of 45 different bees for each experiment.

In experiment 1, we tested bee choices to a *single dangerous feeder* (with *V. velutina*) *and two safe feeders:* a control (no insect) and a feeder with a butterfly that is harmless to bees [38] to control for potential bee aversion to a harmless big insect. All feeders contained 30% sucrose solution. We predicted that bees would avoid the dangerous feeder and prefer the two safe feeders equally.

In experiment 2, we tested bee choices to feeders with *different danger levels*: a big predator (*V. tropica*), a smaller predator (*V. velutin*a), and no hornet (control). All feeders contained 30% sucrose solution. We predicted that individuals would visit dangerous feeders less often, according to the degree of risk.

Finally, we tested the effects of multiple forage qualities and danger levels. In experiment 3 part A, feeders contained 15%, 30% or 45% w/w sucrose solution, but no predators. In experiment 3 part B, we used the same sucrose concentrations, but associated *higher reward with higher risk*. The 15% sucrose solution had no predator and the 30% and 45% sucrose feeders had the small hornet (*V. velutina*) and the big hornet (*V. tropica*), respectively. We predicted that bees would visit dangerous feeders less often, but would factor patch quality (sugar concentration) in their decisions.

### Statistics

We used a Wilcoxon Signed Ranks test to compare the number of hornets of each species *hunting and capturing bees* on flowers. All other data met parametric assumptions as determined through residual analyses. To analyze *heat-balling responses*, we used a Univariate Repeated-Measures Analysis of Variance [48]. *The colony labor allocation* data were also analyzed with a Univariate Repeated-Measures ANOVA. This analysis examines the behavior of colonies, treating each colony as an individual responding to the treatments and we therefore explicitly tested for a colony effect. Because there were no significant colony effects in any of these experiments (see below), we did not test for colony effects in the individual choice experiments. To examine *individual bee choices*, we calculated the proportion of visits made by each bee to each feeder, arcsin square root transformed these proportions [49], and used a Univariate Repeated-Measures ANOVA. In experiment 3B, nine out of 45 bees did not complete all 10 visits. We included these bees in our analysis to provide a symmetric comparison with the colony-level experiments in which bees could also choose not to return. We use Tukey-Kramer Honestly Significant Difference (HSD) Post-Hoc tests, which provide a conservative estimate for pairwise comparisons among treatments [48] and report significant differences as *P*<0.05, following standard conventions [50].

## Results

### Heat-balling experiment

Honey bees colonies attacked both hornet species, which responded aggressively, and attacked the bigger species in higher numbers (Fig. 1A). The mass of *V. tropica* (1.44±0.12 g) is significantly greater than the mass of *V. velutina* (0.36±0.04 g, ANOVA *F*_1,28_ = 1144.61, *P*<0.0001). Significantly more bees balled the bigger hornet (*F*_1,26_ = 844.75, *P*<0.0001): 276.8±29.6 and 62.0±10.4 bees balled *V. tropica* (big hornet) and *V. velutina* (smaller hornet), respectively. Thus, the 4 fold more massive hornet species, *V. tropica*, posed a threat that elicited a 4.6 fold stronger defensive response from colonies.

### Hunting at flowers

Both hornet species attempted to attack *A. cerana* foraging on natural flowers (Fig. 1B). Significantly more (34 fold more) *V. velutina* than *V. tropica* hornets attacked *A. cerana* (Wilcoxon test, *χ* ^2^_1_ = 14.94, *P* = 0.0001, Fig. 1B). For *V. velutina*, 6% of attacks resulted in a kill, the successful captures of a bee. However, we did not observe any (0%) successful *V. tropica* kills (Wilcoxon test, *χ* ^2^_1_ = 6.20, *P* = 0.012). This may have been due to the lower number of *V. tropica* attacks (0.4±0.7 per 30 min) compared to V. *velutina* (13.7±0.6.2 per 30 min). *Apis cerana* foragers also appeared far more wary when foraging in the presence of *V. tropica*, a much bigger hornet (Fig. 1) that should be more visually conspicuous than *V. velutina*.

### Effects on colony foraging allocation

**Figure 2:**
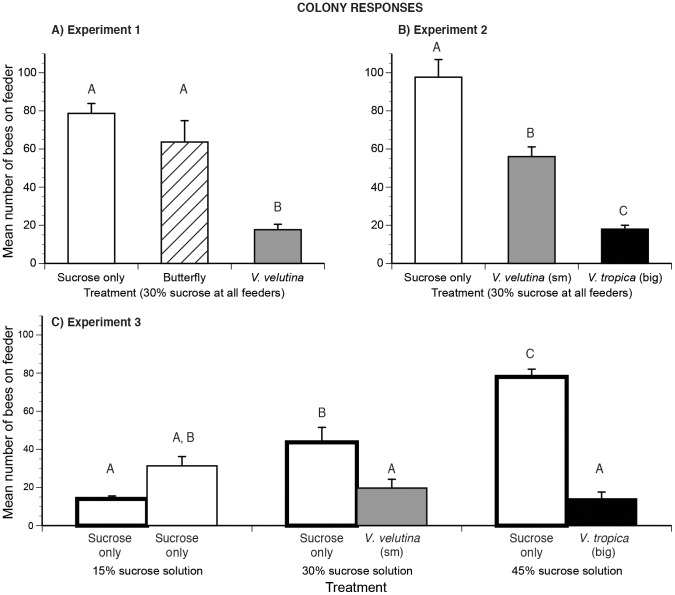
How bee colonies allocate foraging among food sources with different food qualities and levels of predator danger. Sucrose-only feeders are shown as white bars, the butterfly control as a striped bar and the small (sm) and big hornet species as gray and black bars, respectively. Standard error bars are shown. In each graph, different letters indicate significant differences. We show the mean number of foragers at the feeder arrays in (A) experiment 1, (B) experiment 2, and (C) experiment 3 parts A and B. In experiment 3 part A, feeders had sucrose only (bars with thick lines). Part B used the same range of sucrose concentrations, but with the indicated hornet species at the higher sucrose concentrations.

For experiment, 1, there is a significant effect of treatment (*F*_2,4_ = 13.27, *P* = 0.017) and no significant effect of colony (*F*_2,4_ = 0.11, *P* = 0.90). Colonies recruited, on average, 3.2 fold more foragers than were trained and allocated equal numbers of foragers to both safe feeders (Fig. 2A). However, significantly fewer bees fed at the dangerous feeder with the *V. velutina* (small hornet) than at safe feeders with the butterfly or the no-predator control (Tukey-Kramer HSD test, *P*<0.05, Fig. 2A).

In experiment 2, there is also a significant effect of treatment (*F*_2,4_ = 35.70, *P* = 0.003) and no effect of colony (*F*_2,4_ = 0.60, *P* = 0.59). Each colony recruited, on average, 3.4 fold more foragers than were trained. As predicted, colonies allocated the most foragers to the safe control feeder, an intermediate number to the feeder with the small hornet, and the fewest to the feeder with the big hornet. All pairwise comparisons are significantly different (Tukey-Kramer HSD test, *P*<0.05, Fig. 2B).

In experiment 3 (parts A and B), there is likewise a significant effect of treatment (*F*_5,10_ = 22.43, *P*<0.0001, Fig. 2C) and no significant effect of colony (*F*_5,10_ = 0.12, *P* = 0.89). In experiment 3A (sucrose only, no hornets) each colony recruited an average of 2.7 fold more bees than were trained and allocated significantly more labor to richer feeders (all pairwise comparisons significant, Tukey-Kramer HSD test, *P*<0.05). In experiment 3B, each colony recruited an average of 1.3 fold more foragers than were trained, and allocated equal numbers of foragers among all feeders. Comparisons between experiments 3A and 3B show that colonies did not alter their labor allocations to the 15% feeder (always safe). However, colonies significantly decreased the number of foragers allocated to the richer 30% and 45% feeders when predators were added to these feeders (Tukey-Kramer HSD test, *P*<0.05). Thus, when given a choice between different food qualities, colonies did not reallocate more labor to the poor 15% feeder when predators were present at the richer feeders. Colonies were risk-averse and reduced the number of foragers allocated to all dangerous feeders, even though they provided richer rewards (Fig. 2C).

### Effects on individual foraging choice

**Figure 3:**
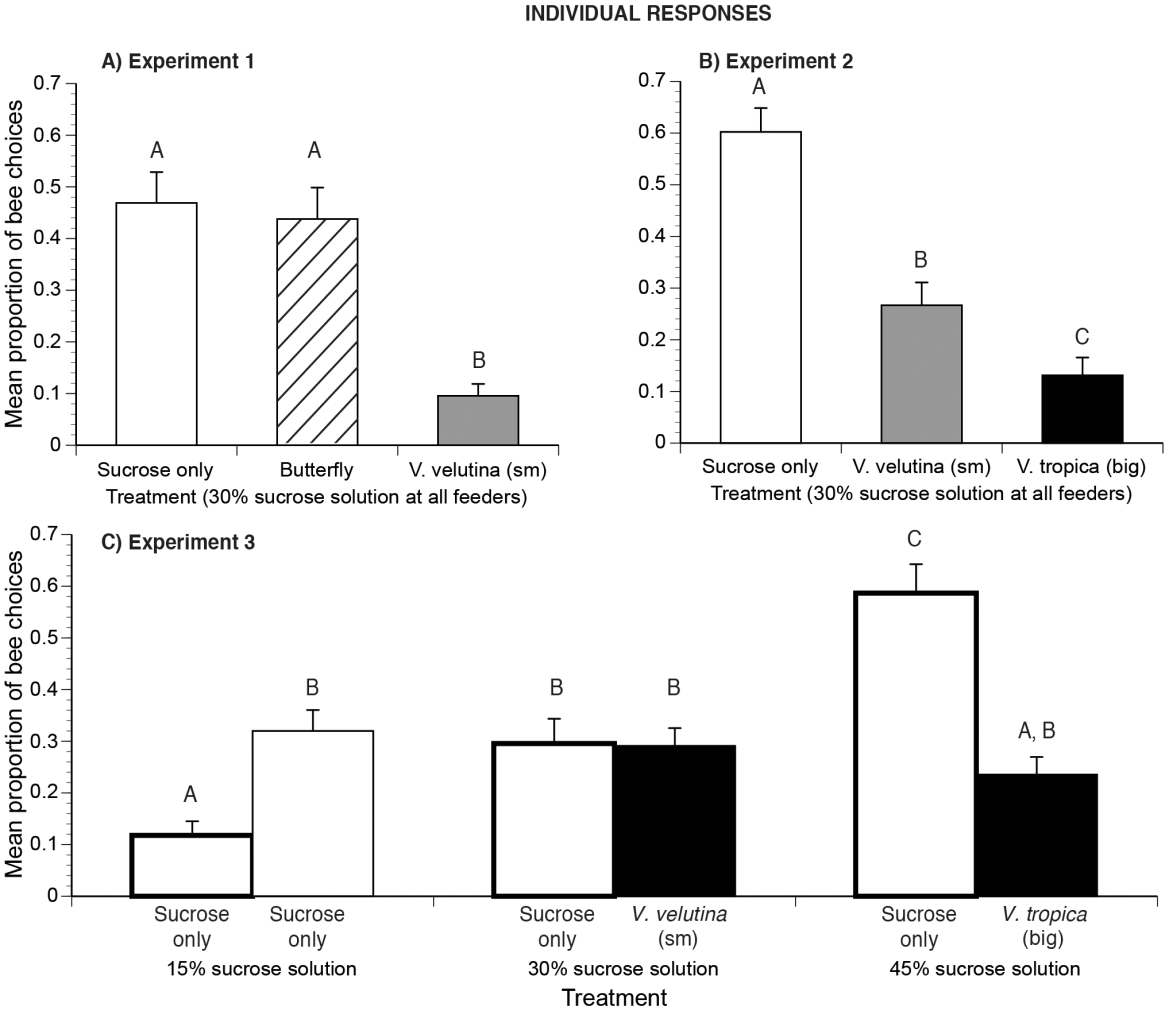
Effects of food quality and predator danger on individual forager choices. Bar patterns and error bars as in Fig. 2. Different letters indicate significant differences. We show the proportion of bee choices for the feeders in (A) experiment 1, (B) experiment 2, and (C) experiment 3 parts A and B. In experiment 3 part A, feeders had sucrose only (bars with thick lines). Part B used the same range of sucrose concentrations, but with the indicated hornet species at the higher sucrose concentrations.

For experiment 1, there is a significant effect of treatment (*F*_2,88_ = 10.00, *P*<0.0001, Fig. 3A). Significantly more bees choose the control and butterfly feeders over the *V. velutina* (small hornet) feeder. There was no significant difference between choices for the control or butterfly feeders (Tukey-Kramer HSD test, *P*<0.05).

For experiment 2, there is a significant effect of treatment (*F*_2,88_ = 24.46, *P*<0.0001, Fig. 3B). Significantly more bees choose the safe feeder over the dangerous feeders. All pairwise comparisons are significantly different (Tukey-Kramer HSD test, *P*<0.05).

For experiment 3 (parts A and B), there is a significant effect of treatment (*F*_5,175_ = 9.53, *P*<0.0001, Fig. 3C). In experiment 3A (sucrose only, no hornets), there is a significant effect of sucrose concentration such that significantly more bees chose the richer feeders (all pairwise comparisons significant, Tukey-Kramer HSD test, *P*<0.05). In experiment 3B, individuals altered their choices and visited all feeders with equal frequency (Fig. 3C). This mirrors colony responses (Fig. 2C).

However, comparisons of parts A and B of experiment 3 reveal that the addition of predators significantly decreased visits to the richest and most dangerous feeder (45% sucrose with big hornet) and commensurately increased visits to poorest but safe feeder (15% sucrose, no predators, Tukey-Kramer HSD test, *P*<0.05). The small hornet at the 30% sucrose feeder *did not* decrease individual visitation to this feeder. Individuals were therefore more risk tolerant than colonies, which reduced foraging for all dangerous feeders (Fig. 2C). In addition, when predators were added to the feeder array, individuals visited the safe and lowest quality feeder significantly more often (Fig. 3C), but colonies did not (Fig. 2C).

### Effects on individual feeding duration

**Figure 4:**
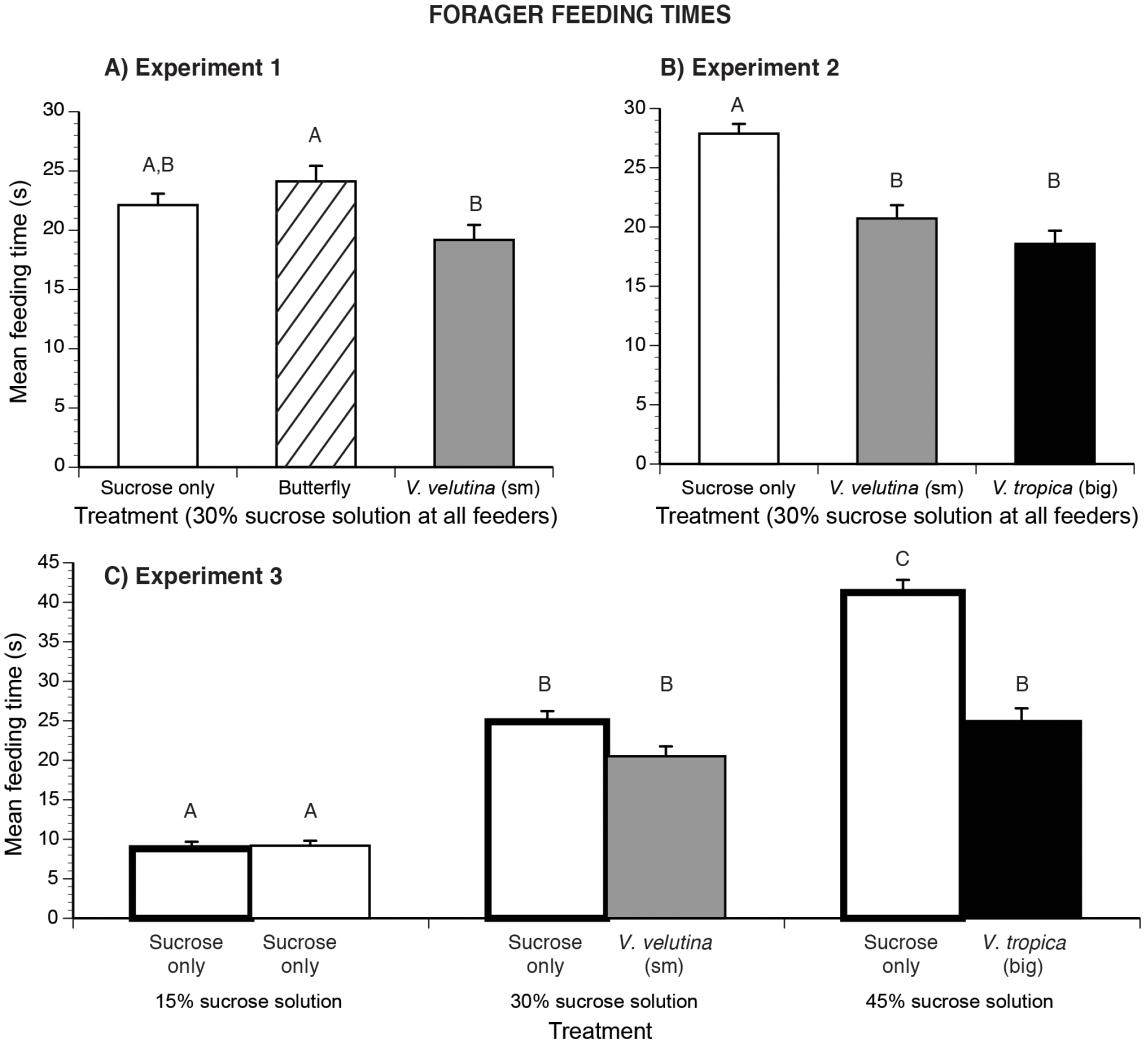
Effects of food quality and predator danger on individual feeding time. Bar patterns and standard error bars as in Fig. 2. Different letters indicate significant differences. Mean feeding times at the feeder arrays in (A) experiment 1, (B) experiment 2, and (C) experiment 3 parts A and B. In experiment 3 part A, feeders had sucrose only (bars with thick lines). Part B used the same range of sucrose concentrations, but with the indicated hornet species at the higher sucrose concentrations.

In experiment 1, there is a significant effect of treatment (*F*_2,58_ = 3.87, *P* = 0.026, Fig. 4A). Bees spent the most time feeding at the butterfly and no-predator control feeders and the least amount of time feeding at the *V. velutina* (small hornet) feeder. Only feeding durations between the butterfly- and *V. velutina*- feeders are significantly different (Tukey-Kramer HSD test, *P*<0.05).

In experiment 2, there is also a significant effect of treatment (*F*_2,58_ = 26.76, *P*<0.0001, Fig. 4B). Bees spent significantly more time feeding at the safe feeder as compared to the feeders with predators. There is no significant difference between feeding times at the feeders with hornets (Tukey-Kramer HSD test, *P*<0.05).

In experiment 3 (parts A and B), there is a significant overall effect of treatment (*F*_5,115_ = 78.84, *P*<0.0001, Fig. 4C). In experiment 3A, bees spent more time feeding on richer feeders (all pairwise comparisons significant, Tukey-Kramer HSD test, *P*<0.05). When predators were added (experiment 3B), bees spent equal time feeding at the two richest (and dangerous) feeders and significantly less time feeding at the poorest but safe feeder (Fig. 4C, Tukey-Kramer HSD test, *P*<0.05).

Comparing between experiments 3A and 3B reveals that bees spent the same amount of time feeding at the 15% feeders in both experiments. The presence of small hornet did not alter feeding time at the 30% feeder. However, the presence of the big hornet significantly decreased the average feeding time at the 45% sucrose feeder. Foragers spent 0.6 fold less time imbibing sucrose when the big hornet was added to the 45% feeder (Fig. 4C).

## Discussion

In our foraging choice experiments, hornets did not kill bees. Thus, the colony- and individual-level bee responses illustrate the strength of non-consumptive predator effects: how fear of prey can strongly and significantly alter foraging even for rich food sources. This is the first detailed demonstration of how fear elicited in a complex natural scenario, multiple predator species and patch qualities, affects individual and collective pollinator foraging behavior. Both big (*V. tropica*) and smaller (*V. velutina*) hornet species attacked *A. cerana* foraging on *Cuphea balsarnona* flowers (Fig. 1B). Based upon bee attack and avoidance behavior, colonies and individuals treated the big hornet as a greater threat than the smaller hornet. The bigger hornet, *V. tropica*, elicited 4.5 fold more attacks from colonies compared to the smaller hornet, *V. velutina* (Fig. 1A). Our foraging experiments confirmed that colony- and individual-level vigilance corresponded to the threat posed by these predators. Colonies and individuals exhibited greater fear (avoidance) of the bigger predator than the smaller predator (Figs. 2,3) and spent less time feeding at dangerous than at safe locations (Fig. 4). When the feeders offered different reward levels (15%, 30%, or 45% sucrose solution w/w), colony and individual foraging was distributed as expected [51], favoring higher sugar concentrations. However, when balancing food quality (sucrose concentration) against multi-predator threats, colonies and individuals differed. Colonies (Fig. 2C) exhibited greater risk aversion than individuals (Fig. 3C). Colonies reduced the number of foragers visiting the intermediate quality feeder when the small hornet was added (55% reduction, Fig. 2C). However, individuals did not alter visitation to this feeder when the small hornet was added (Fig. 3C). Overall, colonies decreased the number of foragers by half when the feeder array became dangerous (Fig. 2C). Individuals were less sensitive: 80% returned to complete all foraging trips when we added hornets.

### Predator threat levels

Significantly more bees were involved in the balling of the bigger hornet (*V. tropica*) than the smaller hornet (*V. velutina*). This is the first data quantifying *A. cerana* balling of *V. tropica*, but we can compare our *V. velutina* results with another study. On average, we counted 62.0±10.4 bees in *V. velutina* balls, similar to the 77.3±23.0 bees reported in Tan et al. [37] and somewhat less than the number of bees involved in balling *V. velutina* and *V. magnifica* (168±52 bees/ball, data includes both hornet species) reported by Abrol [33] in *A. cerana* colonies in northern India. *Apis cerana* foragers were much more wary of the big predator (*V. tropica*, Figs. 2,3,4) and, as expected [52], this big fierce predator was rarer at our floral patch. In 6% of attacks, *Vespa velutina* succeeded in capturing bees, a lower rate than the 32% success rate of another aerial predator, beewolves [15], but higher than the success rates of sit-and-wait predators like crab spiders: 1.6% and 4.8% *Misumena calycina* attacks on bumble bees and other Hymenoptera [24]. At the floral patch, *V. tropica* did not succeed in killing any bees, but it was present in lower numbers and made fewer attacks. Bees strongly avoided this predator (Figs. 2, 3, and 4), which should be visible from a greater distance due to its much larger size (Fig. 1). Increased prey vigilance can reduce successful predation [8]. This may have contributed to the low success rate of *V. tropica* and shows that lethal effects are not the sole driver of prey behavior.

### Prey responses

Our choice experiments manipulated the level of prey danger, representing different patches in a landscape of fear [11]. Both colony- and individual-level responses matched when all feeders presented the same reward quality (30% sucrose solution). Prey avoidance behavior was not simply due to the presence of a big insect on a feeder. Bees did not avoid the harmless butterfly (Figs. 2A, 3A) or spend less time feeding in the butterfly's presence (Fig. 4A) as compared to their behavior on the no-predator feeder. Colonies and individuals avoided hornets, avoiding the bigger hornet more than the smaller hornet (Figs. 2B, 3B) and spent commensurately less time feeding on more dangerous feeders (Fig. 4B). Such evasion could have colony fitness consequences. In the ant, *Lasius pallitarsis*, colonies preferred a higher quality food patch, but decreased foraging for this patch when the danger of predation increased and subsequently experienced decreased colony growth [53].

Many animals will make patch choice decisions that involve energy-predator tradeoffs [54]. Attack by a simulated crab spider on a high-reward flower caused foragers to switch to the lower reward flower [55]. In addition, predators can hunt in patches preferred by prey. Crab spiders preferred to hunt on milkweed inflorescences that offered more nectar and which were more frequently visited by bee pollinators [41], [42]. We therefore varied feeder quality (15%, 30%, or 45% sucrose solution) such that the highest reward corresponded to the highest risk (largest predator).

Colonies and individuals responded by changing their behavior, though in different ways. With predators added, individuals increased their preferences (Fig. 3C) for the only safe feeder (15% sucrose), but colonies did not (Fig. 2C). Colonies exhibited more risk aversion than individuals. Colonies significantly decreased the number of foragers visiting both dangerous feeders (Fig. 2C). Individuals significantly reduced their preferences only for the highest danger and highest reward feeder, but not for the intermediate reward and lower risk feeder (Fig. 3C). Thus, individuals evidently found this intermediate feeder to be worth the risk posed by the smaller hornet when the other choices were a risk-free but poor resource and a more dangerous but richer resource. Colony foraging allocation arises from individual foraging choices and recruitment [56]. The more risk-averse behavior exhibited by the *A. cerana* colonies likely arose from reduced recruitment to the dangerous feeders. In a different species of honey bee, (*A. mellifera*) foragers reduce their recruitment efforts by dancing less for dangerous food sources [45].

With respect to feeding durations, models predict a strong dependence between predation risk and patch residence times in small mammals [54]. Gerbils collect more seeds from sheltered as compared to exposed patches where they are more visible to owls [57]. We observed the same effect in bees. In choices between patches of equal quality, individuals spent equal amounts of time feeding at safe feeders and significantly less time at dangerous feeders, although only the butterfly vs. small hornet comparison was significant (Fig. 4A). In this experiment, small hornet presence reduced bee feeding time by 13% relative to the no-predator control and 20% compared to the butterfly feeder. These relatively small reductions may explain why only the 20% difference was significant.

In the absence of predators, individual feeding time should be correlated to sugar concentration (Fig. 4C) because of nectar viscosity effects and, potentially, reward level. Nectar viscosity increases with sugar concentration [58], [59], and bee imbibing times increase for more viscous solutions, even when sugar concentration is held constant [60]. In addition, some social bees collect larger loads if the nectar has a higher sugar concentration [61]. Higher reward levels may therefore increase feeding duration. Whether *A. cerana* collects larger loads of richer nectar is unknown. *Apis mellifera* foragers carry a constant load of nectar, regardless of sucrose concentration [62], and thus foragers spend more time imbibing more concentrated sucrose solutions because of increased solution viscosity, not reward level. In our experiment, foragers decreased their feeding time only for the most concentrated sugar solution when the big predator was added.

### Effects of fear

We demonstrate two classic effects of fear: changing prey foraging durations and space use. Predator presence reduced the bees spent collecting food by 17–33% (Fig. 4A,B) and elicited bee avoidance, decreasing visitation to dangerous feeders by 55–79% (Figs. 3A,B). These results accord with a diverse animal literature [16]. Redshank birds spend less time foraging in saltmarshes on days with high predation risk from sparrowhawks [63]. Wild herbivores in the African savanna prefer low tree density areas where predators are easier to detect [64].

Our study also illuminates what is less well understood: the role of aerial predators on pollinator behavior, and how fear affects mass recruiting superorganisms in which recruitment can amplify the effects of fear [45]. Research on bee predators has focused on sit-and-wait predators such as crab spiders [65]. However, aerial predators, like hornets, can capture bees in flight and may therefore have a larger spatial influence than sit-and-wait predators, which are more localized. Such aerial insectivores may reduce plant fitness. Flowers of *Hypericum fasciculatum* received fewer pollinator visits near ponds with more dragonfly predators [66].

Finally, we show that colony foraging, the result of mass recruitment, was more affected by fear than individual foraging choice. Mass recruitment is a key foraging strategy of highly social bees [56], [67], which are also important pollinators in ecosystems around the world [68]–[70]. Thus, understanding the emergent effects of fear at the superorganism level, how colonies differ in fearful behavior from their individual members, will give us a better understanding of how fear has shaped the evolution of bee foraging and how the effects of fear can be amplified at the colony level, thereby exerting a wider effect on a vital ecosystem service, pollination.

## References

[1] Shurin JB, Clasen JL, Greig HS, Kratina P, Thompson PL (2012) Warming shifts top-down and bottom-up control of pond food web structure and function. Philosophical Transactions of the Royal Society B: Biological Sciences 367: 3008–3017 doi:10.1890/08-2254.1 10.1098/rstb.2012.0243 PMC3479752 23007089

[2] Wilby A, Orwin KH (2010) Herbivore species richness, composition and community structure mediate predator richness effects and top-down control of herbivore biomass. Appl Microbiol Biotechnol 87: 87–97 doi:10.1007/s00253-010-2573-8 23292455 10.1007/s00442-012-2573-8

[3] Knight TM, Chase JM, Hillebrand H, Holt RD (2006) Predation on mutualists can reduce the strength of trophic cascades. Ecol Letters 9: 1173–1178 doi:10.1111/j.1461-0248.2006.00967.x 10.1111/j.1461-0248.2006.00967.x 17040319

[4] Buchmann SL, Nabhan GP (1997) The forgotten pollinators. Island Press. 1 pp.

[5] Tepedino VJ (1980) The importance of bees and other insect pollinators in maintaining floral species composition. Great Basin Naturalist Memoirs 3: 139–150.

[6] Suttle KB (2003) Pollinators as mediators of top-down effects on plants. Ecol Letters 6: 688–694 doi:10.1046/j.1461-0248.2003.00490.x

[7] Lima SL (1998) Nonlethal effects in the ecology of predator-prey interactions. BioScience 48: 25–34 doi:10.2307/1313225

[8] Brown JS, Laundré JW, Gurung M (1999) The ecology of fear: optimal foraging, game theory, and trophic interactions. Journal of Mammalogy 80: 385–399.

[9] Ives AR, Dobson AP (1987) Antipredator behavior and the population dynamics of simple predator-prey systems. Am Nat 130: 431–447.

[10] Sih A (1992) Prey uncertainty and the balancing of antipredator and feeding needs. American Naturalist 1052–1069.

[11] Laundré JW, Hernández L, Ripple WJ (2010) The landscape of fear: ecological implications of being afraid. Open Ecology Journal 3: 1–7.

[12] Blumstein DT (2006) Developing an evolutionary ecology of fear: how life history and natural history traits affect disturbance tolerance in birds. Animal Behaviour 71: 389–399.

[13] Preisser EL, Bolnick DI, Benard MF (2005) Scared to death? The effects of intimidation and consumption in predator–prey interactions. Ecology 86: 501–509 doi:10.1890/04-0719

[14] Gonçalves-Souza T, Omena PM, Souza JC, Romero GQ (2008) Trait-mediated effects on flowers: artificial spiders deceive pollinators and decrease plant fitness. Ecology 89: 2407–2413. 18831161 10.1890/07-1881.1

[15] Dukas R (2005) Bumble bee predators reduce pollinator density and plant fitness. Ecology 86: 1401–1406.

[16] Romero GQ, Antiqueira PAP, Koricheva J (2011) A meta-analysis of predation risk effects on pollinator behaviour. PLoS ONE 6: e20689 doi:10.1371/journal.pone.0020689 21695187 10.1371/journal.pone.0020689 PMC3113803

[17] Sih A, Englund G, Wooster D (1998) Emergent impacts of multiple predators on prey. Trends Ecol Evol (Amst) 13: 350–355. 21238339 10.1016/s0169-5347(98)01437-2

[18] Lima SL (1992) Life in a multi-predator environment: some considerations for anti-predatory vigilance. Ann Zool Fennici 29: 217–226.

[19] Dukas R, Morse DH (2005) Crab spiders show mixed effects on flower-visiting bees and no effect on plant fitness components. Ecoscience 12: 244–247.

[20] Dukas R, Morse DH (2003) Crab spiders affect flower visitation by bees. Oikos 101: 157–163.

[21] Dukas R (2001) Effects of perceived danger on flower choice by bees. Ecol Letters 4: 327–333.

[22] Reader T, Higginson AD, Barnard CJ, Gilbert FS (2006) The effects of predation risk from crab spiders on bee foraging behavior. Behavioral Ecology 17: 933–939.

[23] Robertson IC, Maguire DK (2005) Crab spiders deter insect visitations to slickspot peppergrass flowers. Oikos 109: 577–582.

[24] Morse DH (1979) Prey capture by the crab spider *Misumena calycina* (Araneae: Thomisidae). Oecologia 39: 309–319. 28309174 10.1007/BF00345442

[25] Jha S, Kremen C (2013) Resource diversity and landscape-level homogeneity drive native bee foraging. Proceedings of the National Academy of Sciences 110: 555–558 doi:10.1073/pnas.1208682110 10.1073/pnas.1208682110 PMC3545746 23267118

[26] Tan K, Radloff SE, Li JJ, Hepburn HR, Yang MX, et al (2007) Bee-hawking by the wasp, *Vespa velutina*, on the honeybees *Apis cerana* and *A. mellifera* . Naturwissenschaften 94: 469–472 Available: http://www.springerlink.com/index/10.1007/s00114-006-0210-2. 17235596 10.1007/s00114-006-0210-2

[27] Morse DH (1986) Predatory risk to insects foraging at flowers. Oikos 223–228.

[28] Schmitz OJ, Krivan V, Ovadia O (2004) Trophic cascades: the primacy of trait-mediated indirect interactions. Ecol Letters 7: 153–163 doi:10.1111/j.1461-0248.2003.00560.x

[29] Wilson EE, Holway DA (2010) Multiple mechanisms underlie displacement of solitary Hawaiian Hymenoptera by an invasive social wasp. Ecology 91: 3294–3302. 21141190 10.1890/09-1187.1

[30] Thomson JD (1989) Reversal of apparent feeding preferences of bumble bees by aggression from *Vespula* wasps. Can J Zool 67: 2588–2591.

[31] Hanna C, Foote D, Kremen C (2012) Invasive species management restores a plant-pollinator mutualism in Hawaii. J Appl Ecol 50: 147–155 doi:10.1111/1365-2664.12027

[32] Burgett M, Akratanakul P (1982) Predation on the western honey bee, *Apis mellifera* L., by the hornet, *Vespa tropica* (L.). Psyche: A Journal of Entomology 89: 347–350 doi:10.1155/1982/37970

[33] Abrol DP (2006) Defensive behaviour of *Apis cerana* F. against predatory wasps. Journal of Apicultural Science 20: 39–46.

[34] Seeley TD, Seeley RH, Akratanakul P (1982) Colony defense strategies of the honeybees in Thailand. Ecological Monographs 52: 43–63.

[35] Tan K, Li H, Yang MX, Hepburn HR, Radloff SE (2010) Wasp hawking induces endothermic heat production in guard bees. Journal of Insect Science 10: 1–6 doi:10.1673/031.010.14102 21073346 10.1673/031.010.14102 PMC3016720

[36] Matsuura M, Yamane S (1990) Biology of the vespine wasps. Springer Verlag. 1 pp.

[37] Tan K, Yang M-X, Wang Z-W, Li H, Zhang ZY, et al (2011) Cooperative wasp-killing by mixed-species colonies of honeybees, *Apis cerana* and *Apis mellifera* . Apidologie 43: 195–200 doi:10.1007/s13592-011-0098-5

[38] Tan K, Wang Z, Li H, Yang S, Hu Z, et al (2012) An “I see you” prey–predator signal between the Asian honeybee, *Apis cerana*, and the hornet, *Vespa velutina* . Animal Behaviour 83: 879–882 doi:10.1016/j.anbehav.2011.12.031

[39] Guan Huang Y (2005) Harm of introducing the western honeybee *Apis mellifera* L. to the Chinese honeybee *Apis cerana* F. and its ecological impact. Acta Entomologica Sinica 3: 015.

[40] Corlett RT (2001) Pollination in a degraded tropical landscape: a Hong Kong case study. J Trop Ecol 17: 155–161 doi:10.1017/S0266467401001109

[41] Morse DH (1986) Foraging behavior of crab spiders (*Misumena vatia*) hunting on inflorescences of different quality. American Midland Naturalist 116: 341–347.

[42] Morse DH, Fritz RS (1982) Experimental and observational studies of patch choice at different scales by the crab spider *Misumena vatia* . Ecology 63: 172–182.

[43] Anderson C, Ratnieks FLW (1999) Worker allocation in insect societies: coordination of nectar foragers and nectar receivers in honey bee (*Apis mellifera*) colonies. Behavioral Ecology and Sociobiology 46: 73–81.

[44] Tan K, Yang MX, Radloff SE, Hepburn HR, Zhang ZY, et al (2008) Dancing to different tunes: heterospecific deciphering of the honeybee waggle dance. Naturwissenschaften 95: 1165–1168 doi:10.1007/s00114-008-0437-1 18688588 10.1007/s00114-008-0437-1

[45] Abbott KR, Dukas R (2009) Honeybees consider flower danger in their waggle dance. Animal Behaviour 78: 633–635.

[46] Ranabhat NB, Tamrakar AS (2009) Study on seasonal activity of predatory wasps attacking honeybee *Apis cerana* Fab. colonies in southern belt of Kaski District, Nepal. Journal of Natural History Museum 23: 125–128.

[47] Giurfa M (2004) Conditioning procedure and color discrimination in the honeybee *Apis mellifera* . Naturwissenschaften 91: 228–231 doi:10.1007/s00114-004-0530-z 15146270 10.1007/s00114-004-0530-z

[48] Lehman A, O'Rourke N, Hatcher L, Stepanski EJ (2005) JMP for basic univariate and multivariate statistics: a step-by-step guide. Cary, NC: SAS Institute Inc. 1 pp.

[49] Zar JH (1984) Biostatistical analysis. Englewood Cliffs: Prentice-Hall.

[50] Wendorf CA (2012) Part IV: statistics in APA style. Statistics for psychologists: calculating and interpreting basic statistics using SPSS. University of Wisconsin, Stevens Point. pp. 1–6.

[51] Waddington KD, Gottlieb N (1990) Actual vs perceived profitability: A study of floral choice of honey bees. J Insect Behav 3: 429–441 doi:10.1007/BF01052010

[52] Colinvaux PA (1978) Why big fierce animals are rare: an ecologist's perspective. Princeton Academic Press. 1 pp.

[53] Nonacs P, Dill LM (1990) Mortality risk vs. food quality trade-offs in a common currency: ant patch preferences. Ecology 1886–1892.

[54] Lima SL (1998) Stress and decision making under the risk of predation: recent developments from behavioral, reproductive, and ecological perspectives. Advances in the Study of Behavior 27: 215–290.

[55] Jones EI, Dornhaus A (2011) Predation risk makes bees reject rewarding flowers and reduce foraging activity. Behavioral Ecology and Sociobiology 65: 1505–1511 doi:10.1007/s00265-011-1160-z

[56] Dyer FC (2002) The biology of the dance language. Annu Rev Entomol 47: 917–949 doi:10.1146/annurev.ento.47.091201.145306 11729095 10.1146/annurev.ento.47.091201.145306

[57] Kotler BP, Brown JS, Hasson O (1991) Factors affecting gerbil foraging behavior and rates of owl predation. Ecology 72: 2249–2260.

[58] Kim W, Gilet T, Bush JWM (2011) Optimal concentrations in nectar feeding. Proceedings of the National Academy of Sciences 108: 16618–16621 doi:10.1073/pnas.1108642108 10.1073/pnas.1108642108 PMC3189050 21949358

[59] Kingsolver JG, Daniel TL (1995) Mechanics of food handling by fluid-feeding insects. Regulatory mechanisms in insect feeding. Boston, MA: Regulatory Mechanisms in Insect Feeding. pp. 32–73. doi:10.1007/978-1-4615-1775-7_2.

[60] Borrell BJ (2006) Mechanics of nectar feeding in the orchid bee *Euglossa imperialis*: pressure, viscosity and flow. Journal of Experimental Biology 209: 4901–4907 doi:10.1242/jeb.02593 17142679 10.1242/jeb.02593

[61] Roubik DW, Buchmann SL (1984) Nectar selection by *Melipona* and *Apis mellifera* (Hymenoptera: Apidae) and the ecology of nectar intake by bee colonies in a tropical forest. Oecologia 61: 1–10 doi:10.1007/BF00379082 28311379 10.1007/BF00379082

[62] Wells PH, Giacchino J (1968) Relationship between the volume and the sugar concentration of loads carried by honeybees. Journal of Apicultural Research 7: 77–82.

[63] Hilton GM, Ruxton GD, Cresswell W (1999) Choice of foraging area with respect to predation risk in redshanks: the effects of weather and predator activity. Oikos 295–302.

[64] Riginos C, Grace JB (2008) Savanna tree density, herbivores, and the herbaceous community: bottom-up vs. top-down effects. Ecology 89: 2228–2238. 18724733 10.1890/07-1250.1

[65] Higginson AD, Ruxton GD, Skelhorn J (2010) The impact of flower-dwelling predators on host plant reproductive success. Oecologia 164: 411–421 doi:10.1007/s00442-010-1681-6 20563603 10.1007/s00442-010-1681-6

[66] Knight TM, McCoy MW, Chase JM, McCoy KA, Holt RD (2005) Trophic cascades across ecosystems. Nature 437: 880–883 doi:10.1038/nature03962 16208370 10.1038/nature03962

[67] Nieh JC (2004) Recruitment communication in stingless bees (Hymenoptera, Apidae, Meliponini). Apidologie 35: 159–182 doi:10.1051/apido:2004007

[68] Partap U (2011) The pollination role of honeybees. Honeybees of Asia. Berlin: Springer Verlag. pp. 227–255.

[69] Garibaldi LA, Steffan-Dewenter I, Winfree R, Aizen MA, Bommarco R, et al (2013) Wild pollinators enhance fruit set of crops regardless of honey bee abundance. Science 339: 1608–1611 doi:10.1126/science.1230200 23449997 10.1126/science.1230200

[70] Potts SG, Biesmeijer JC, Kremen C, Neumann P, Schweiger O, et al (2010) Global pollinator declines: trends, impacts and drivers. Trends Ecol Evol (Amst) 25: 345–353 doi:10.1016/j.tree.2010.01.007 20188434 10.1016/j.tree.2010.01.007

